# Perceived Criticism and Family Attitudes as Predictors of Recurrence in Bipolar Disorder

**DOI:** 10.32872/cpe.4617

**Published:** 2022-03-31

**Authors:** Claudia Lex, Martin Hautzinger, Thomas D. Meyer

**Affiliations:** 1 Department of Psychiatry, Villach General Hospital, Villach, Austria; 2 Department of Psychology, University Klagenfurt, Klagenfurt, Austria; 3 Department Clinical Psychology and Psychotherapy, Eberhard Karls University Tuebingen, Tuebingen, Germany; 4 McGovern Medical School, Louis A. Faillace, MD, Department of Psychiatry and Behavioral Sciences, University of Texas HSC at Houston, Houston, TX, USA; Philipps-University of Marburg, Marburg, Germany

**Keywords:** bipolar disorder, cognitive behavioral therapy, expressed emotion, perceived criticism, illness course, family, psychotherapy

## Abstract

**Background:**

Bipolar disorder (BD) is a highly recurrent psychiatric condition. While combined pharmacological and psychosocial treatments improve outcomes, not much is known about potential moderators that could affect these treatments. One potential moderator might be the quality of interpersonal relations in families, for example, familial attitudes and perceived criticism.

**Method:**

To explore this question we conducted a post-hoc analysis that used an existing data set from a previous study by our group that compared cognitive behavioral therapy (CBT) and supporting therapy (ST) in remitted BD. In the present study, we used Cox proportional hazard models.

**Results:**

We found that the relatives’ ratings of criticism predicted the likelihood of depressive recurrences, especially in the ST condition. The patients’ ratings of negative familial attitudes predicted the risk of recurrences in general, irrespective of the therapy condition.

**Conclusion:**

These results suggest that it might be important to assess perceived criticism and familial attitudes as potential moderators of treatment outcome in BD.

## Highlights

- Patients with BD had an increased risk for depressive recurrences when their relatives had rated themselves as highly critical towards the patients. This was only true for patients who attended an unspecific therapy instead of CBT.
- Patients with BD had an increased risk for depressive recurrences when they thought that their relatives had negative attitudes towards them.
- There was no significantly increased risk for manic recurrences in relation or criticism or negative familial attitudes.

Bipolar disorder (BD) is a mental health condition characterized by depressive and hypomanic or manic episodes. While individuals experiencing BD can remit, it is considered a life-long condition and over 50% of patients with BD suffer at least one recurrence within two years (Perlis et al., 2006; Tohen et al., 2003). Furthermore, functional impairments at work, home, or school, and in interpersonal relations often persist beyond symptomatic states of the disorder and despite medication (Gitlin & Miklowitz, 2017). These findings on long-term outcomes of BD have encouraged experts to develop and evaluate psychosocial and psychological therapies adjuvant to medication. The combination of psychological and pharmacological treatments overall improves the long-term outcome in BD (Miklowitz & Scott, 2009; Swartz & Swanson, 2014) but the evidence is mixed. A recent network analysis showed that the evidence is stronger for some therapies such as Family Focused Therapy (FFT) or Cognitive Behavior Therapy (CBT) than others, but that these findings should be balanced against evidence that dropping out of CBT is more likely than for FFT, and that efficacy varies depending on the outcome such as recurrence, depressive or manic symptoms (Miklowitz et al., 2021). For example, FFT seems to protect against recurrences, especially in families with greater levels of impairment (Kim & Miklowitz, 2004). CBT, however, was specifically associated with stabilizing depressive symptoms (Miklowitz et al., 2021). In general, more studies are needed to determine under what circumstances which form of psychological therapy is most effective.

One potential factor or moderator of outcome in BD could be the quality of interpersonal relations, because similar to other psychiatric disorders (e.g. Grover & Dutt, 2011; Hooley & Teasdale, 1989; Weintraub et al., 2017) it has been suggested that characteristics of familial relations may also predict outcome in bipolar depression (Johnson et al., 2016). In regard to BD, criticism expressed by families when interacting with their ill relative predicted hospital admissions (Scott et al., 2012) and relapse (Rosenfarb et al., 2001). Also, high expressed emotion, which is a construct that is characterized by critical comments, hostility, and emotional over-involvement that family members express towards an affected relative (Kavanagh, 1992; Vaughn & Leff, 1976), predicted relapses as does a communication style called ‘negative affective style’ (Miklowitz et al., 1988; O’Connell et al., 1991). Finally, two studies found that perceived criticism and expressed emotion were specifically associated with depressive rather than with manic recurrences (Kim & Miklowitz, 2004; Yan et al., 2004).

Most of the before mentioned studies looked at the natural course of BD. In order to examine if perceived criticism and hostile/critical attitudes influence the effect of CBT on recurrences in BD, we reanalyzed data previously collected in a randomized controlled trial (Meyer & Hautzinger, 2012). In this study individual CBT and supportive therapy (ST) were administered to patients with remitted BD. CBT was manual-based including cognitive and behavioral strategies, techniques to prevent relapse, and coping strategies for symptoms (Basco & Rush, 1996). ST was less structured and followed a client-centered approach. In the original study (Meyer & Hautzinger, 2012), it was found that the relapse rates did not significantly differ between the two therapy groups in the long run. However, a higher number of prior mood episodes and a lower number of attended therapy sessions were associated with less time to relapse in both groups, indicating that other potential factors shared by both groups influenced outcome. Based on the evidence cited above, we hypothesized that higher levels of negative familial attitudes and perceived criticism expressed by the patients with BD and their relatives could be such a moderator of outcome.

## Method

### Participants

**Figure 1:**
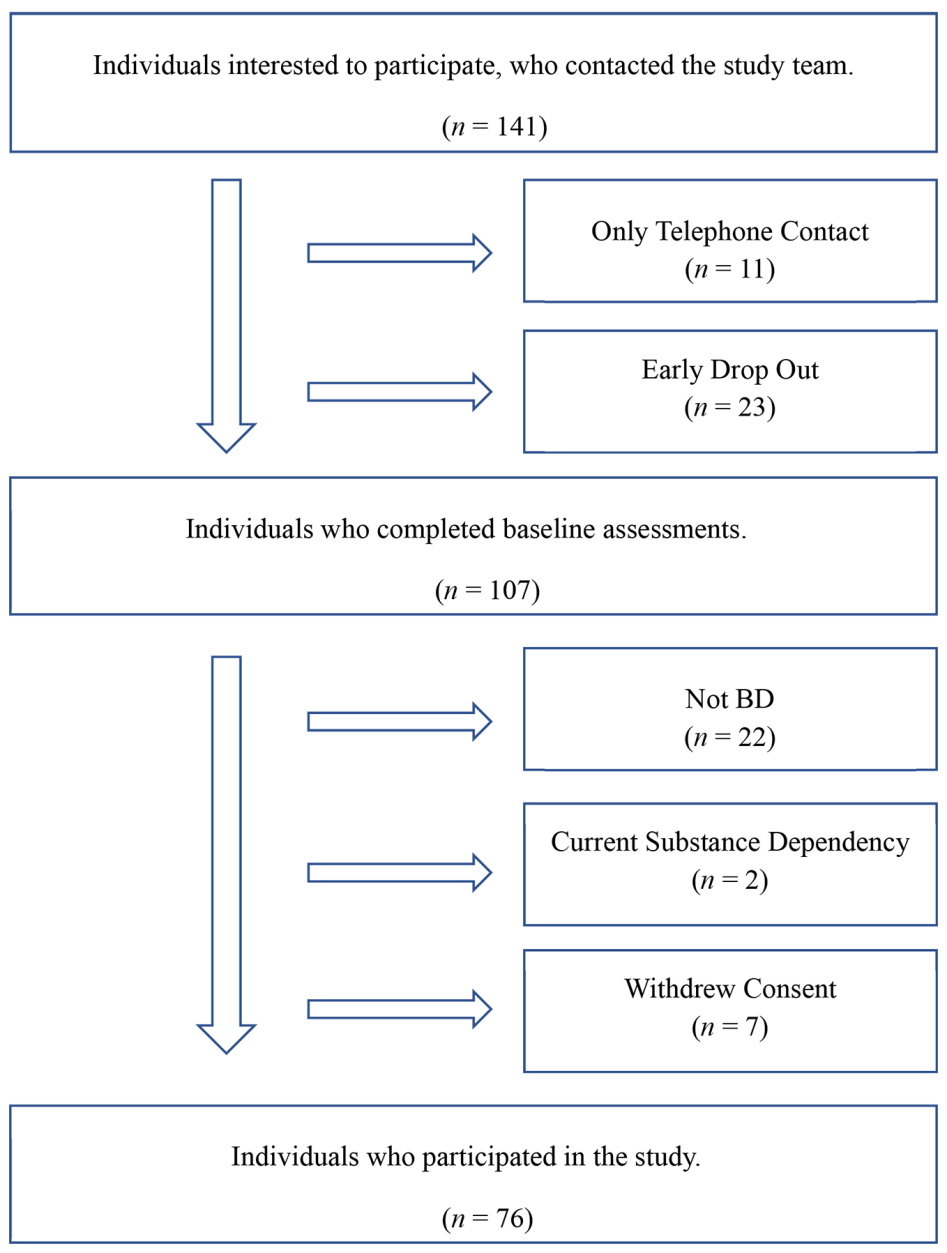
Flow Chart of the Recruitment Process *Note.* Not BD = Individuals who were not diagnosed with BD.

Initially, 141 individuals who were interested to participate in a study of psychological treatment for BD contacted our study team. They were either referred by local hospitals, psychiatrists or were self-referrals due to public information in newspapers, brochures, or radio. Sixty-five individuals were excluded (Figure 1), therefore, the present paper reports data relating to clinical course and attitudes of 76 participants who were randomized for a study on psychotherapy for BD (Meyer & Hautzinger, 2012).

Inclusion criteria were a diagnosis of BD, age between 18 and 65, informed consent to the present study, and adherence to their usual psychiatric treatments. Participants with severe manic or depressive symptoms, i.e. scores > 20 on the Bech-Rafaelsen Melancholia Scale (BRMS; Bech, 2002) or > 20 on the Bech-Rafaelsen Mania Scale (BRMAS; Bech et al., 1978), were excluded. Also, participants with comorbid substance dependency requiring detoxification and/or the presence of current psychotic symptoms could not participate in the present study. We obtained informed consent that included the consent to send a questionnaire to their spouse, or if single or divorced to their partner or closest relative (e.g. mother).

### Procedures and Measurements

First, the participants were in a baseline screening session. They gave informed consent, were administered clinical interviews (e.g., SCID-I and SCID-II), and completed self- and observer-rated measures (see for further details: Meyer & Hautzinger, 2012). Then they were randomized either to an individual CBT or supportive therapy (ST), which both contained individual 20 sessions over 9 months. The CBT followed a structured manual similar to the manual by Basco and Rush (1996), which included relapse prevention plans, coping strategies, and interpersonal skills. In the ST a client centered approach was adopted focusing on whatever topics the individuals brought into the sessions. All sessions were video-taped. Qualified therapists who were at least in a 1-year postgraduate training led the sessions. In addition, all therapists attended a 2-day workshop relating to CBT and ST therapy. Raters who were blind to group allocation assessed conducted assessments at month 0, 3, 6, 9, 12, and 24 during the trial. Information on recurrences was obtained by using repeatedly the SCID-I modules for mood episodes during the follow-up but also by monitoring hospitalizations, clinical notes, and mood diaries of the participants.

#### Family Attitude Scale (FAS)

The FAS (Kavanagh et al., 1997) contains 30 items covering 4 key aspects of critical attitudes among close family members: criticism, hostility, anger, and warmth. The items are rated on a 5 point scale ranging from *always* (4) to *never* (0), therefore scores may range between 0 to 120. Higher scores reflect higher levels of critical familial attitudes. We used two versions of the FAS, one for patients (FAS-P; e.g., “He/she thinks, that I am a real burden”) and one for relatives (FAS-R; e.g., “He/she is a real burden”). The FAS-P, therefore, reflects how the patient perceives the attitudes of his/her relative, while the relative reports in the FAS-R how he/she feels about the patient and what he/she thinks about the patient. In order to obtain a German version, the senior author translated the original English version, and then a native English speaker did the backtranslation. The inconsistencies were discussed and finally removed. To our knowledge, the German FAS has not been formally validated, but we published high internal consistencies for the FAS-P (Cronbach's α = 0.94) and for the FAS-R Cronbach's α = 0.95; Lex et al., 2019).

#### Perceived Criticism Measure (PCM)

The rating on a 10 point scale of the question "How critical is your relative of you?" has been used as a valid indicator of overall criticism in families (Hooley & Miklowitz, 2017; Renshaw, 2007). Therefore, in the PCM-P (Hooley & Teasdale, 1989) we asked the patients to rate the question “How critical has he/her been of you?”. Parallel, the relatives self-rated their level of criticism with the question “How critical have you been of him/her?” (PCM-R). Although there is no recommended cutoff, higher scores reflect higher levels of criticism and a score above 6 raises concern about an increased relapse risk (Masland & Hooley, 2015). Information about correlates of the German PCM scale can be found in Lex et al. (2019).

#### Beck Depression Inventory (BDI)

The BDI (Beck et al., 1961) is a self-report questionnaire measuring the severity of depression. Participants rate 21 items that correspond to depressive symptoms on a four-point scale from 0 to 3. Scores above 9 reflect mild, and scores above 18 reflect moderate depression. In the present study, we used the validated German version with comparable psychometric properties compared to the English version (Brieger et al., 2007; Hautzinger et al., 1994).

#### Self Rating Mania Inventory (SRMI)

The SRMI (Shugar et al., 1992) is a 47-item self-rating instrument that assesses manic and hypomanic symptoms. It can be used to assess acute symptoms or residual symptoms in remitted states. In the present study, we asked the participants to focus on the previous month when rating their (hypo)manic symptoms. Scores above 14 reflect a high probability of acute mania. The SRMI shows a good internal consistency (Cronbach's α = 0.94) and high retest reliabilities between 0.79 and 0.93 (Shugar et al., 1992).

#### Bech Rafaelsen Melancholia Scale (BRMS)

The observer-based BRMS (Bech, 2002; Smolka & Stieglitz, 1999) has 11 items that relate to depressive symptoms and is used to rate the severity of depression. The rating for each items ranges from 0 (no symptom) to 4 (severe). A sum score ≤ 14 indicates no or doubtful depression, scores between 15 and 20 indicate mild depression, 21–28 indicate moderate depression, and scores above 28 reflect severe depression (Lam et al., 2005).

#### Bech Rafaelsen Mania Scale (BRMAS)

The BRMAS (Bech et al., 1978) has 11 items and the observer rates the presence of manic symptoms on a scale from 0 (not present) to 4 (severe). Parallel to the BRMS, scores range between 0 and 44, and scores < 14 suggest no or doubtful mania, scores between 15 and 20 indicate mild mania, and scores above 20 are interpreted as moderate to severe mania (Lam et al., 2005). The BRMAS shows good interrater reliabilities between 0.80 and 0.95 (e.g., Bech, 2002). The BRMAS is often combined with the BRMS to cover the full range of bipolar symptoms (Rossi et al., 2001).

### Statistical Methods

Hierarchical Cox proportional hazard models were used to assess the relapse risk for depression in relation to the patients’ and the relatives’ assessments of familial attitudes and perceived criticism. The potential covariates were therapy condition (CBT vs. ST; Block 1), attitudes (FAS or PCM scores; Block 2), and the interaction between therapy and attitudes (block 3). When looking at the recurrence risk for (hypo)manic events, SRMI scores were entered at Block 1, the subsequent blocks were the same as before. SRMI scores were included, because in a previous analysis we found that the only baseline clinical variable that predicted recurrence of manic episodes was the level of subthreshold self-reported manic symptoms (Bauer et al., 2017). With less than 5% of the corresponding z-scores being greater than 1.96, there were no outliers for the FAS and PCM measures. There were no substantial bivariate correlations between predictors (see Table 1) indicating that there was no problem with multicollinearity (Field, 2013). In addition, bivariate listwise correlations and independent *t*-Tests were used. The significance level was set at 5% for all statistical procedures, exact *p* values and effect size values will be displayed.

**Table 1:** Bivariate Listwise Pearson Correlations Between Predictors, FAS, and PCM Measures

*N* = 76	FAS-P	PCM-P	FAS-R	PCM-R
Therapy Condition	.05	.19	.17	.04
FAS-P		.48**	.47**	.29*
PCM-P			.30*	.49**
FAS-R				.40**

*Note*. FAS-P = Family Attitude Scale rated by patients; FAS-R = Family Attitude Scale rated by relatives; PCM-P = Perceived Criticism Scale rated by patients; PCM-R = Perceived Criticism Scale rated by relatives.

*p < .05. **p < .01.

## Results

### Demographics

The participants’ mean age was 43.96 (*SD* = 11.81) and included 38 women. Thirty-two individuals were single, 31 were married, and 13 were divorced. Sixty individuals were diagnosed with BD-I, and 16 were diagnosed with BD-II. Based on the SCID-I, all participants were in full remission; looking at rating scales, most patients had scores below 15 on the BRMS (93.4%) and the BRMAS (98.7%). Table 2 displays demographical and clinical data of the participants. The participants of CBT and ST did not differ significantly on age, gender, clinical course of BD, and time until first relapse (Meyer & Hautzinger, 2012). Also, conducting independent *t*-tests revealed that scores on the FAS-P, *t*(66) = -.66, *p* = .51 and the PCM-P, *t*(66) = -1.47, *p* = .15, for the patients did not differ significantly between the two treatment conditions. Similarly, the scores in the FAS-R, *t*(62) = -.90, *p* = .37 and the PCM-R, *t*(61) = -.18, *p* = .85, were not significantly different in relatives of the patients who had been randomly assigned to CBT and ST.

**Table 2:** Means (M) and Standard Deviations (SD) of Patients With BD Who Received Either CBT or ST

Variable	CBT		ST	
	*M*	*SD*	*M*	*SD*
Age	44.40	11.00	43.53	12.72
BDI	13.53	9.23	11.03	7.60
BRMS	6.08	4.70	5.55	5.24
SRMI	17.65	10.98	19.00	11.19
BRMAS	2.34	3.69	1.03	2.56
N of prior episodes	11.18	15.17	10.13	10.61
Age of onset	26.63	9.24	29.84	12.44
Weeks until relapse	54.95	46.36	50.08	51.64
Patient FAS	39.63	19.58	40.10	15.58
Patient PCM	4.69	2.49	5.47	1.81
Relative FAS	33.08	15.99	36.68	16.18
Relative PCM	4.88	2.31	4.97	1.64

*Note.* BDI = Beck Depression Inventory; BRMS = Bech Rafaelsen Melancholia Rating Scale; BRMAS = Bech Rafaelsen Mania Rating Scale; FAS = Family Attitude Scale; PCM = Perceived Criticism Scale; SRMI = Self-Rating Mania Inventory (Meyer & Hautzinger, 2012).

### Cox Proportional Hazards Models

**Figure 2:**
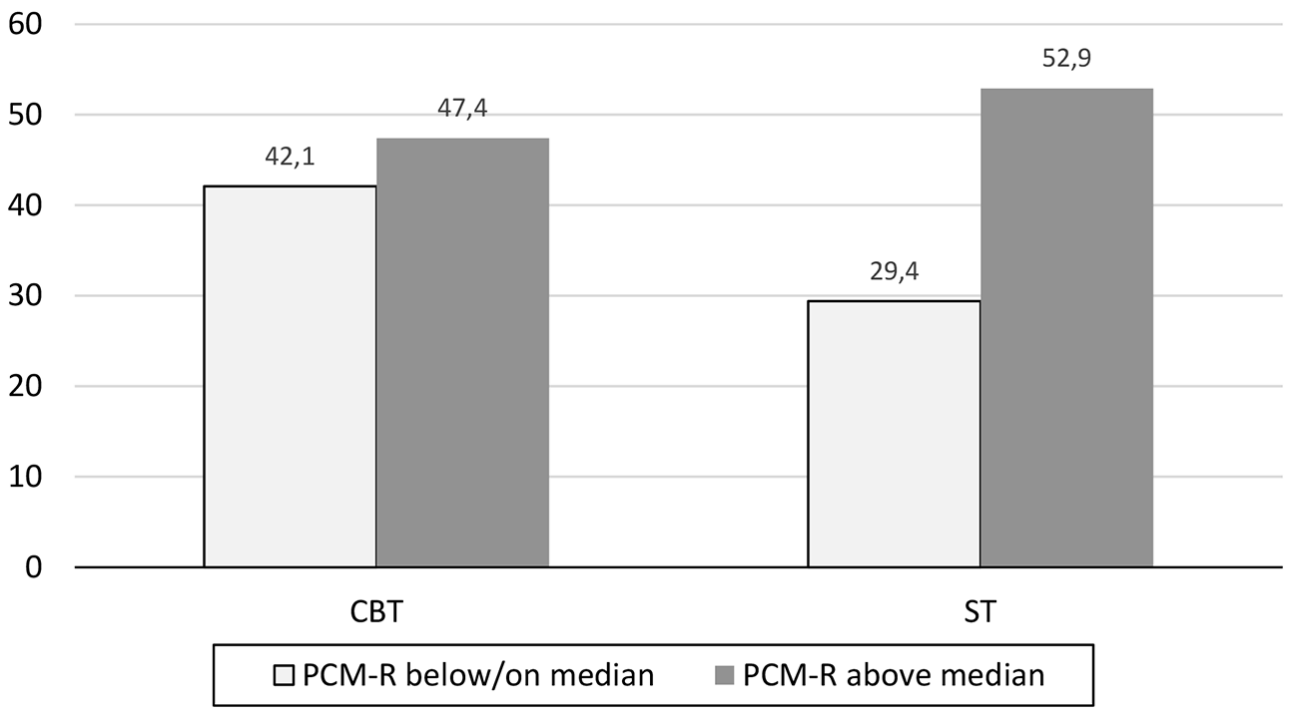
Percentage of Participants With a Depressive Recurrence Whose PCM-R Scores Were Below/On and Above the Median *Note.* CBT = Cognitive Behavioral Therapy; PCM-R = Perceived Criticism Scale rated by relatives; ST = Supportive Therapy.

The Cox proportional hazards model included the two measures of interest (FAS and PCM), the therapy condition (CBT and ST), and their interaction. First, the outcome was defined as recurrence of a depressive episode. Table 3 contains the relevant outcome values of these analyses. Two separate models were calculated: one for patients’ and one for relatives’ scores. Although, the overall model for the patients was not significant; χ^2^ = 7.65, *p* = .18, the FAS-P predicted significantly more recurrences of depressive episodes. The overall model for relatives was also not significant, χ^2^ = 6.27, *p* = .28, but PCM-R significantly interacted with therapy group in predicting depressive recurrences. Specifically, increased PCM-R predicted a higher number of depressive recurrences in the ST group but not in the CBT group (Figure 2).

**Table 3:** Cox Proportional Hazards Models Testing FAS and PCM as Predictors of Depressive Recurrence

Variable	*B*	*Wald*	*p*	*HR*	95% CI for HR		χ^2^	*p*
					*LL*	*UL*		
Patients								
Model 1							0.16	.69
Therapy	.14	0.16	.69	1.15	0.57	2.31		
Model 2							6.38	.09
Therapy	.07	0.04	.84	1.08	0.53	2.19		
FAS-P	.03	5.89	.01	1.03	1.01	1.06		
PCM-P	-.14	1.53	.22	0.87	0.70	1.08		
Model 3							7.65	.18
Therapy	-.74	0.54	.46	0.47	0.06	3.49		
FAS-P	.04	2.94	.09	1.04	1.00	1.08		
PCM-P	-.28	2.63	.10	0.76	0.54	1.06		
FAS-P x Therapy	-.01	0.16	.69	0.99	0.94	1.04		
PCM-P x Therapy	.24	1.16	.28	1.27	0.82	1.97		
Relatives								
Model 1							0.36	.55
Therapy	.22	0.36	.55	1.25	0.61	2.57		
Model 2							2.27	.52
Therapy	.27	0.51	.48	1.32	0.62	2.80		
FAS-R	.01	1.19	.28	1.01	0.99	1.04		
PCM-R	.03	0.06	.81	1.03	0.83	1.27		
Model 3							6.27	.28
Therapy	1.82	2.12	.15	6.19	0.53	71.92		
FAS-R	-0.01	0.27	.61	0.99	0.96	1.03		
PCM-R	0.40	3.52	.06	1.50	0.98	2.28		
FAS-R x Therapy	0.03	1.68	.20	1.03	0.98	1.09		
PCM-R x Therapy	-0.52	4.43	.03	0.59	0.37	0.97		

*Note.* B = regression coefficient; FAS-P = Family Attitude Scale rated by patients; FAS-R = Family Attitude Scale rated by relatives; PCM-P = Perceived Criticism Scale rated by patients; PCM-R = Perceived Criticism Scale rated by relatives; HR = hazard ratio; SRMI = Self Rating Mania Scale; Wald = Wald test.

When the outcome was defined as recurrence of (hypo)manic episodes, the overall models for patients (χ^2^ = 11.89, *p* = .07) and for relatives (χ^2^ = 7.34, *p* = .29) were not significant. In both models, the score of the SRMI was the only significant predictor of manic recurrences (Table 4).

**Table 4:** Cox Proportional Hazards Models Testing FAS and PCM as Predictors of (Hypo)Manic Recurrence

Variable	*B*	*Wald*	*p*	*HR*	95% CI for HR		χ^2^	*p*
					*LL*	*UL*		
Patients								
Model 1							5.11	.02
SRMI	.04	4.92	.03	1.04	1.01	1.08		
Model 2							6.01	.05
SRMI	.04	5.00	.03	1.04	1.01	1.08		
Therapy	.44	1.05	.31	1.55	.67	3.56		
Model 3							10.01	.04
SRMI	.05	6.63	.01	1.05	1.01	1.09		
Therapy	.37	.74	.39	1.45	.62	3.37		
FAS-P	-.01	.66	.42	.99	.95	1.02		
PCM-P	-.12	1.10	.29	.88	.70	1.11		
Model 4							11.89	.07
SRMI	.06	8.12	.004	1.07	1.02	1.11		
Therapy	-.30	.05	.82	.74	.06	9.18		
FAS-P	-.05	3.23	.07	.95	.90	1.01		
PCM-P	.05	.07	.77	1.05	.73	1.51		
FAS-P x Therapy	.06	2.56	.11	1.06	.99	1.13		
PCM-P x Therapy	-.29	1.26	.26	.75	.45	1.24		
Relatives								
Model 1							4.24	.04
SRMI	.04	4.13	.04	1.04	1.00	1.08		
Model 2							4.86	.09
SRMI	.04	4.25	.04	1.04	1.00	1.08		
Therapy	.37	.70	.40	1.44	.61	3.40		
Model 3							6.50	.17
SRMI	.05	4.93	.03	1.05	1.00	1.08		
Therapy	.41	.85	.36	1.51	.63	3.64		
FAS-R	.001	.01	.95	1.00	.97	1.03		
PCM-R	-.13	1.38	.24	.88	.70	1.09		
Model 4							7.34	.29
SRMI	.04	4.48	.03	1.05	1.00	1.09		
Therapy	1.27	.86	.35	3.58	.24	52.50		
FAS-R	.004	.02	.89	1.00	.95	1.06		
PCM-R	-.03	.02	.90	.97	.58	1.62		
FAS-R x Therapy	-.01	.06	.80	.99	.93	1.06		
PCM-R x Therapy	-.12	.18	.67	.89	.51	1.55		

*Note.* B = regression coefficient; FAS-P = Family Attitude Scale rated by patients; FAS-R = Family Attitude Scale rated by relatives; PCM-P = Perceived Criticism Scale rated by patients; PCM-R = Perceived Criticism Scale rated by relatives; HR = hazard ratio; SRMI = Self Rating Mania Scale; Wald = Wald test.

## Discussion

The present study explored whether negative familial attitudes and perceived criticism predicted recurrences in euthymic individuals with BD who attended individual CBT or ST. In general, there was no significant difference in risk of recurrence between the two groups (Meyer & Hautzinger, 2012), but the present post-hoc exploration showed that the relatives’ rating of their own perceived criticism towards the patient influenced the likelihood of depressive recurrences to a greater extent in the ST than in the CBT condition. In addition, the patients’ perception of the family climate was related to the risk of depressive recurrences. There was no significant link between indicators for the familial climate and the risk for manic recurrences. These results are in line with previous studies that report familial criticism was linked to depressive relapse and symptoms but not to mania (Kim & Miklowitz, 2004; Yan et al., 2004).

At first sight, the interaction between treatment condition and self-rated perceived criticism of the relatives towards the patient (PCM-R) remains puzzling. However, the wording of the item for relatives refers to how much they see themselves being critical of the patients. The data therefore suggests that admitting more critical comments on side of the relatives increased risk for depressive recurrences specifically in the ST group, while it did not make a difference in the CBT group. One goal of the manual-based CBT was to help patients to differently communicate and solve problems which often includes how to react to perceived criticism. Although this is speculative, this perhaps helped to protect against being criticized or differently to react to perceived criticism. For example, the patients might learn to attribute critical remarks to their relatives’ mood or the specific situation instead to their own person. In ST, the patients did not specifically learn communication or coping skills, therefore pre-treatment differences in actual or perceived criticism by the relative might still have had the same effect on risk of recurrence as having had no treatment, while CBT helped to attenuate the effect of this factor. While the latter is a potential explanation of the differential effect, it remains unclear why the relatives’ but not the patients’ perception of criticism had an impact on recurrence rates. This is puzzling because a) PC measures were administered at baseline, i.e. before the therapy sessions started, b) the PC of patients and relatives were positively correlated at baseline, and c) both therapies were done in an individual and not in a couple or family setting. In addition, while it is an intriguing idea that individual CBT might be effective in families with a hostile and critical climate, it is important to keep mind that these conclusions are exploratory and based on post-hoc analyses.

Regardless of the condition, patients who perceived their familial climate as more hostile had an increased risk for depressive recurrences. This is in line with previous studies that found that expressed emotions were linked to more depressive symptoms (Kim & Miklowitz, 2004) and recurrences (Yan et al., 2004). Those studies, however, used observer-based assessments based on frequency counts of critical and hostile behavior while we assessed the familial climate with questionnaires. The mostly used version of the FAS is self-rated by the patient and asks for specific thoughts, behaviors and feelings expressed by the relative towards the patient (e.g., “He/she loses his/her temper with me”; “He/she thinks I am real burden”; “He/she feels very close to me”). The patient-rated FAS was found to be related to relapse in patients with psychosis (Pourmand et al., 2005), and its content rather taps into hostility and criticism than to emotional overinvolvement, which is considered as one of the key factors of expressed emotion (Kavanagh et al., 1997). In the present study, we also used a relatives’ version of the FAS, and we found that it did not significantly predict the risk of recurrences. Although observer-rated measures, e.g., the Camberwell Family Interview (Leff & Vaughn, 1985), are regarded as the gold standard to assess the familial climate (Hooley & Parker, 2006), our results suggest that the patient-rated FAS could be a sensible instrument to tap intrafamilial hostility and criticism and to predict depressive recurrences in BD. It is essential to keep in mind that in the FAS the patient reports his/her perception of the family member’s attitudes and feelings, while the relative reports how he/she actually feels and what he/she thinks.

Interestingly, the relatives’ one-item measure PCM interacted with therapy group to predict relapses, while the patients’ FAS predicted relapses regardless of the treatment condition. First, this result emphasizes the importance to assess criticism and hostility in both interaction partners, because it is still not clear how the reciprocity of interactions relate to hostility, criticism and expressed emotion (Hooley & Gotlib, 2000). For example, hostility expressed by a relative’s remark could be escalated or descaled depending on the response by the patient. Second, patients’ actual perceptions of the attitudes are important, because the patient might or might not identify the hostility and criticism expressed by the relative (Yan et al., 2004). While the FAS and PCM share variance, they do not assess identical constructs (Lex et al., 2019). While perceived criticism, whether rated by the patient or relative, is fairly specific, the FAS encompasses more general negative attitudes within the family beyond critical comments. Possibly, in patients this perception of criticism can be better measured by ratings of a range of specific behaviors, feelings and thoughts, i.e., FAS, while in relatives the one-item measure PCM might be sufficient.

This is one of the few studies in which criticism and hostile familial attitudes, two key elements of expressed emotion, were rated by the affected individuals and their relatives themselves instead by observers. Although the PCM and the FAS have empirical evidence to predict relapse similar to the more time consuming interviews or observations of actual family interactions (Chambless & Blake, 2009; Hooley & Parker, 2006; Kavanagh et al., 1997), relying solely on self-reports is a limitation of the study. Also, emotional overinvolvement as a key factor of expressed emotion was not assessed. We also received information from only one relative who might not be the one who necessarily was the most critical or most relevant person for the patient. Some studies suggest that the kind of relation between the relative and patient might play a crucial role (Hooley, 2007). Finally, as mentioned before, these were post hoc analyses, therefore the study was probably not powered to test for these interactions which is probably reflected in the non-significant overall models.

### Conclusions

Despite this limitations, we found preliminary evidence that perceived criticism and familial attitudes in individuals with BD and their relatives were associated with an increased risk for depressive recurrences. Specifically, the relatives’ self-rated own criticism towards the patient affected outcome in the ST group more that in the CBT group, and an overall negative family climate as perceived by patients predicted outcome regardless of the therapy conditions, when it referred to depressive recurrences. The different results for the one-item measure PCM and the FAS support the idea that these instruments share some variance but do not assess identical constructs. While this was a first step to explore the usefulness of self-ratings of family attitudes and expressed emotion in BD, our results encourage the idea to use such questionnaires that are easy to administer in clinical practice to assess the familial climate (Chambless & Blake, 2009; Masland & Hooley, 2015). These preliminary results also stress the need for future studies to explore in more detail the potential moderating role of expressed emotions in different psychological therapies (Miklowitz & Chambless, 2015) and specifically in different stages of BD.
